# Preferences for innovations in healthcare delivery models in the Swiss elderly population: a latent class, choice modelling study

**DOI:** 10.1093/eurpub/ckae004

**Published:** 2024-01-18

**Authors:** Anna Nicolet, Clémence Perraudin, Nicolas Krucien, Joël Wagner, Isabelle Peytremann-Bridevaux, Joachim Marti

**Affiliations:** Center for Primary Care and Public Health (Unisanté), University of Lausanne, Lausanne, Switzerland; Center for Primary Care and Public Health (Unisanté), University of Lausanne, Lausanne, Switzerland; Patient-Centered Research, Evidera, London, UK; Department of Actuarial Science, Faculty of Business and Economics (HEC), and Swiss Finance Institute, University of Lausanne, Lausanne, Switzerland; Center for Primary Care and Public Health (Unisanté), University of Lausanne, Lausanne, Switzerland; Center for Primary Care and Public Health (Unisanté), University of Lausanne, Lausanne, Switzerland

## Abstract

**Background:**

With the increasing number of people affected by multiple chronic conditions, it is essential for public-health professionals to promote strategies addressing patient needs for coordinated care. We aim to explore preference heterogeneity for better-coordinated care delivery models in Swiss older adults, and identify profiles of individuals more open to healthcare reforms.

**Methods:**

A DCE (discrete choice experiment) survey was developed online and on paper for the Swiss adults aged 50+, following best practice. To elicit preferences, we estimated a latent class model allowing grouping individuals with similar preferences into distinct classes, and examined what background characteristics contributed to specific class membership.

**Results:**

The optimal model identified three classes with different openness to reforms. Class 1 (49%) members were concerned with premium increases and were in favour of integrated care structures with care managed by interprofessional teams. Individuals in class 2 (19%) were younger, open to reforms, and expressed the needs for radical changes within the Swiss healthcare system. Class 3 respondents (32%) were strongly reluctant to changes.

**Conclusions:**

Our study goes beyond average preferences and identifies three distinct population profiles, a majority open to reforms on specific aspects of care delivery, a smallest group in favour radical changes, and a third strongly against changes. Therefore, tailored approaches around healthcare reforms are needed, e.g. explaining the role of interprofessional teams in coordinating care, electronic health records and insurance premium variation.

## Introduction

Increasing number of people with multiple chronic conditions poses considerable challenges in care organization, planning, and costs for public-health policies and healthcare systems worldwide. With the epidemiological transition from acute to chronic conditions and an increasing life expectancy, public-health professionals and policymakers must respond to a growing number of complex patients with frequent transitions between care settings. In Switzerland, chronic conditions account for more than 80% of total outpatient healthcare expenditures.^1^ Moreover, with continuously increasing healthcare expenditures (>12% of GDP spent on healthcare), the country is facing increasing burden on households due to high out-of-pocket payments and community-rated premiums,^2–4^ while healthcare resources are not always allocated efficiently.^1^^,^^2^ Therefore, a recurring topic of political discussions is the enhancement of equity in financing and the development of alternative models of healthcare delivery (e.g. models of coordinated care).^3^^,^^5–7^ With such models, care is provided by multiple healthcare professionals, requiring good communication and information exchange (e.g. access to patient history, care plan, etc.), aiming to deliver holistic and appropriate services reflecting patients’ needs.^8^^,^^9^ In a recent expert consultation at the Federal level, care coordination was broadly discussed, and several coordination-improving measures were suggested: definition of patient pathways, enabling all providers to communicate better and develop a common understanding of their role; or promotion of networks with direct financial incentives for better coordination.^6^ However, implementing countrywide innovations may be challenging due to the complex, decentralized and fragmented organization and financing of the Swiss healthcare system.^2^^,^^3^^,^^10^ The complexity lies in the governance structure, whereby general healthcare regulations are imposed at the national level, but many more specific decisions are taken at the cantonal level (26 cantons). In addition, health insurance is mandatory in Switzerland but there is high private actor involvement in financing, as the plans are sold by many private companies (>50) in a strongly regulated market. Additional details on the Swiss healthcare system can be found elsewhere.^8^

An additional challenge to implement large-scale reforms is the Swiss political system that relies on direct democracy. The Swiss population can veto or demand reform through public referenda,^2^ allowing populations to be directly involved in decision making. Therefore, an efficient communication strategy with clear explanations for the population is needed, as most voters may not understand proposals on complex subjects such as healthcare. In Switzerland, where development of innovative health policy implementations has been under heated debates for a long time,^3^^,^^11^ it is critical to identify the population groups that would be willing to accept changes, and to tailor the communication strategies accordingly.

In such context, eliciting population preferences is a key element to increase acceptability of potential policy changes.^12^ Discrete choice experiments (DCE) have been widely used for health policy, planning and resource allocation decisions in healthcare to quantify public preferences and trade-offs for care access and delivery characteristics in countries with different financing and healthcare structures.^13–15^ A DCE asks participants to repeatedly indicate their preferred option among hypothetical scenarios, and after completing a series of trade-offs the researchers can elicit preferences. Among the advantages of the DCEs are the ability to mimic decision-making processes in the real-world settings, visual attractiveness and interactivity for respondents, and relative ease of completion.^16^ There is an increasing trend of DCE use for the development of health policies by analysing the preferences of target groups for different aspects of the reform or intervention, as it allows to model the uptake of new initiatives or inform on the relative importance of aspects comprising a complex scenario.^14^^,^^16^^,^^17^ In the Swiss context, earlier DCE studies quantified trade-offs of the public and service providers between current healthcare insurance plan (status quo) and proposed innovations.^18^^,^^19^ These studies showed that the Swiss population is reluctant to accept changes; therefore, expressing a high attachment to status quo, especially among older age groups.^18–20^

However, the importance of understanding how health treatments, policies or services could be tailored to various population groups has been widely recognized but still understudied, as their opinions and values are not homogenous.^21^ Implementing general recommendations following a ‘one size fits all’ approach may be inefficient and infeasible,^22^ and the interest in exploring population heterogeneity has been growing rapidly among policy makers.^13^^,^^23^ Specifically, in the Swiss context where population is directly involved in the decision making via direct democracy, it is crucial to understand what population subgroups are more open to reforms, and what aspects of the reforms are of higher importance for them.

Therefore, the aim of this study was first to explore heterogeneity of population preferences for alternative better-coordinated models, and then to understand the profiles of Swiss adult population groups who were more open to healthcare reforms.

## Methods

### Selection of attributes

Details on the qualitative development of the survey, attributes and levels are available elsewhere.^22^ In brief, an extensive literature search was first performed yielding an initial set of 33 attributes, followed by multiple rounds of stakeholders’ involvement, to obtain a manageable subset of attributes (*N* = 8). These attributes were tested and refined in focus groups with the general population and patients. A final list of six attributes was validated in an online pilot study (*N* = 301) and includes: access to EMR (electronic medical record), designated care coordinator, access to the specialists, formal compensation for informal care givers, exemption of chronic patients from paying deductibles and/or co-payments, and monthly premium change.

### Survey design

The survey contained three main sections: general introduction and collection of background information, the experimental part (i.e. the DCE), and follow-up questions on health, healthcare use, choice of health insurance, and opinions on the Swiss healthcare system. The details of the study design may be found elsewhere.^8^^,^^22^ The experimental part of the survey consisted of repeated choices between two alternatives and a status quo option, and was presented in a ‘dual response format’.^24^ Specifically, participants were first asked to choose between two hypothetical care models, and next to repeat their choice between the stated preferred option and a status quo (‘my current model’).

We used a D-efficient design and divided a subset of 42 possible choice sets into seven survey versions with six choice questions per version, which were used for subsequent choice modelling, plus one practice task and one consistency test task, which were used to evaluate the quality of the choice data. Details of experimental design are described elsewhere.^22^ An example of a choice set, and a survey version are presented in Supplementary Material.

### Data collection

In total, 3472 individuals from the Swiss population aged 50+ and residing in French-speaking cantons were invited to participate by mail. We focused on population aged 50+ as this subgroup is characterized by the onset of multiple chronic conditions associated with increasing costs, and thus, they are more likely to benefit from new better-coordinated care models. Moreover, as such coordinated models do not exist in its defined form in Switzerland yet, it is important to understand preferences of the target population for the potential of such models, to inform their future design and communication strategy. For respondents under 70 years old, an invitation letter to participate in the online survey, programmed in Qualtrics (Provo, UT, USA), was followed by two reminders. The same procedure was used for potential participants aged 70 years and more, with an option of answering on paper upon request. A telephone hotline was set up to handle participants’ questions and issues with the survey. Participants were rewarded via a lottery to win a CHF 300 cash prize (ca. USD 300).

### Statistical analysis

First, we produced descriptive statistics of study participants: socio-demographic data, health status, healthcare utilization, insurance coverage and opinions on the Swiss health care system. For the DCE section, the outcome of each of the six choice sets was a three-level categorical variable (alternative 1, alternative 2, or current model [status quo]), which can be interpreted as the conditional probability of preferring one alternative over the others given the attribute levels in the choice set.^25^

To investigate preference heterogeneity, we estimated a latent class logit (lclogit, Stata 16.0) model allowing identification of individuals who can be grouped together.^26^ The optimal number of classes is exploratory, not predetermined, and is based on a measure of class differentiation for a range of possible class numbers (in our case 2–7), information criteria AIC (Akaike Information Criteria) and BIC (Bayesian Information Criteria). The model with the lowest AIC/BIC was considered superior to the other models (Supplementary table S1). To obtain a quantitative measure of how well the model did in differentiating several classes of preferences, we computed the average (over all respondents) of the highest posterior probability of class membership.^26^

Next, we described the classes by examining which background characteristics were associated with the probability of a certain class membership. First, each respondent was classified into the class with the highest posterior membership probability. Then, we analysed the associations between class membership and individual characteristics (e.g. socio-demographic, health-related, insurance-related, trust and opinions on Swiss healthcare, and health literacy) using multinomial logit model (mlogit, Stata 16.0).

## Results

### Sample characteristics

Data were collected between March and April 2021. A total of 1385 individuals participated in the survey, 227 surveys were filled in paper format and 1158 online (response rate of 39% for individuals below 70, and 41% for individuals above 70 years old). However, 187 invitations were returned by the post office, e.g. because the recipient could not be found, or the letter was refused. Excluding the respondents who only partially or not at all completed the experimental part, we ended up with 975 fully completed surveys used for analysis, including 118 surveys in paper format. The median time for online completion was 23.9 min; overall 82% passed the consistency test, and 21% systematically chose the status quo option. Median age was 65, there were more males (59%) than females, and 36% reported having 0 diagnosed chronic conditions, while 32.3% reported having been diagnosed with 2+ diseases (table 1).

**Table 1:** Characteristics of the study sample

	All DCE completed
N total	975 (paper 118, online 857)
Socio-demographic characteristics	
Age mean, med, min–max	65.8, 65, 58–73
Males, *N* (%)	571 (58.56%)
Education, *N* (%)	
Compulsory schooling	15 (1.55%)
Apprenticeship	80 (8.25%)
High school diploma	376 (38.76%)
Professional diploma	158 (16.29%)
University, higher education	341 (35.15%)
Income, *N* (%)	
Less than 5000 CHF	306 (31.61%)
5000–7000 CHF	221 (22.83%)
7000–9000 CHF	161 (16.63%)
9000–11000 CHF	96 (9.92%)
Over 11 000CHF	151 (15.60%)
I do not know/I do not want to answer	33 (3.41%)
Marital status, *N* (%)	
Single	116 (11.91%)
Married/Registered partnership	528 (54.21%)
Separated	223 (22.90%)
Widower widow	107 (10.99%)
Living arrangement, *N* (%)	
Alone	285 (29.35%)
Single-parent family	54 (5.56%)
Couple without children	355 (36.56%)
In couple with child(ren)	241 (24.82%)
Other	32 (3.30%)
Professional status, *N* (%)	
Full time employee	278 (28.63%)
Part-time employee	76 (7.83%)
Self-employed	60 (6.18%)
Unemployed	27 (2.78%)
Retirement	476 (49.02%)
Disability insurance (AI)	17 (1.75%)
At home to do household	14 (1.44%)
Other	23 (2.37%)
Health-related characteristics	
Health state, *N* (%)	
Very good	184 (18.87%)
Good	512 (52.51%)
Neither good nor bad	209 (21.44%)
Bad	68 (6.97%)
Very bad	2 (0.21%)
Disease, *N* (%)	
No diseases (reported)	343 (35.99%)
1 disease	296 (30.36%)
2+ diseases^a^	315 (32.31%)
Health insurance and healthcare utilization characteristics	
Insurance model, *N* (%)	
Standard insurance	377 (39.03%)
HMO	30 (3.11%)
Family doctor model	457 (47.31%)
Consultation by telephone beforehand (Telemed)	81 (8.39%)
Other	14 (1.45%)
I do not know	7 (0.72%)
Deductible levels, *N* (%)	
Low (300 CHF or 500 CHF)	648 (66.67%)
Medium (1000 CHF, 1500 CHF or 2000 CHF)	129 (13.33%)
High (2500 CHF)	181 (18.62%)
I do not know	14 (1.44%)
Premium, *N* (%)	
Less than 350 CHF	172 (17.84%)
351–450 CHF	323 (33.51%)
451–550 CHF	325 (33.71%)
More than 550 CHF	129 (13.39%)
I do not know	15 (1.56%)
Do you receive insurance subsidy (Yes), *N* (%)	137 (14.08%)
Do you have complementary insurance (Yes), *N* (%)	744 (77.66%)
Have you ever given up receiving medical services due to financial reasons (Yes), *N* (%)	133 (13.70)
Have you visited any physician within 12 months (Yes), *N* (%)	677 (70.45%)
Have you been hospitalized within 12 months (Yes), *N* (%)	154 (16.04%)
Have you been to the emergency department (Yes), *N* (%)	133 (13.91%)
Have you been admitted to the nursing home within 12 months (Yes), *N* (%)	7 (0.73%)
Received informal care	
Yes	150 (15.59%)
No	812 (84.41%)
Provided informal care	
Yes	342 (35.66%)
No	617 (64.34%)
Health literacy characteristics	
Confidence in the health insurance model chosen, *N* (%)	
Confident	673 (69.17%)
Neither confident nor not confident	233 (23.95%)
Not confident	67 (6.89%)
Experience difficulties in understanding written medical information, *N* (%)	
Never	401 (41.47%)
Sometimes	468 (48.40%)
Often	64 (6.62%)
Always	34 (3.52%)
Opinions and trust in healthcare system in Switzerland	
I think healthcare system in Switzerland:	
Requires no reform	37 (3.83%)
Requires a small number of reforms	556 (57.56%)
Requires substantial reforms	330 (34.16%)
Cannot choose	43 (4.45%)
Is the system when the richest can afford better healthcare than poorest fair?	
Fair	124 (12.8%)
Neither fair nor unfair	210 (21.67%)
Unfair	612 (63.16%)
Cannot choose	23 (2.37%)
Would you be ready to pay more taxes for better medical care for everyone in Switzerland?	
Ready	203 (21.00%)
Neither ready, nor not ready	271 (28.02%)
Not ready	473 (48.92%)
Cannot choose	20 (2.07%)
Support or oppose the system with public fund for obligatory health insurance, N (%)	
Support	658 (68.05%)
Neither in favour nor opposed	154 (15.93%)
Oppose	135 (13.96%)
Cannot choose	20 (2.07%)
Characteristics of the survey completion	
Time to completion (minutes): mean, med, IQR	28.2, 23.9, 17.7–32.4
Passed stability task, *N* (%)	799 (82%)
Passed practice (dominance) task, *N* (%)	774 (79%)
Only chose left or right alternative—all tasks, *N* (%)	64 (6.6%)
Only chose status quo	207 (21%)
Never chose status quo	93 (9.5%)

a Among those: hypertension 67, high cholesterol 55, angina 6, heart failure 14, stroke 2, diabetes 32, COPD 10, osteoporosis 7, arthritis 44, cancer 10, ulcer 2, depression 14, HIV 1.

### Preferences for healthcare models expressed by different population subgroups

#### Estimates from latent classes

The estimation procedure led to a 3-classes solution, with the following distribution of respondents across classes: class 1 (49%), class 2 (19%) and class 3 (32%). The mean highest posterior probability of class membership was 0.92, meaning that the model performed well at distinguishing different underlying taste patterns for the observed choice behaviours. In line with our previous findings,^8^ respondents attached more importance to monthly premium, care coordination, access to EMR and specialist access. However, despite specific similarities across classes (e.g. disutility of premium increase or restriction of access to specialist), there was substantial heterogeneity in preferences across the three classes (table 2).

**Table 2:** Latent class parameter estimates (*N* = 975)

Attribute level	Class 1 Share 49.2%	Class 2 Share 18.9%	Class 3 Share 31.9%
	Coef. (SE)	Coef. (SE)	Coef. (SE)
Access to EMR			
*All healthcare professionals (incl. non-doctors) involved in care, plus health insurance (ref)*			
GP only	−0.36 (0.12)^***^	0.20 (0.22)	0.81 (0.67)
All doctors involved in care	0.11 (0.11)	0.82 (0.23)^***^	0.76 (0.69)
All healthcare professionals (incl. non-doctors) involved in care	0.36 (0.11)^***^	0.46 (0.21)^**^	1.66 (0.70)^**^
Designated care coordinator			
*A referent person from health insurance (ref)*			
None	−0.23 (0.13)^*^	0.68 (0.19)^***^	−1.53 (0.59)^***^
GP	0.55 (0.11)^***^	1.79 (0.24)^***^	0.57 (0.61)
Healthcare professional (non-doc)	0.09 (0.12)	0.88 (0.19)^***^	−1.93 (0.44)^***^
Team	0.66 (0.11)^***^	1.20 (0.18)^***^	−1.31 (0.55)^**^
Access to the specialist			
*Free (ref)*			
Only via GP who is a gatekeeper	−0.60 (0.10)^***^	−0.28 (0.17)^*^	−2.27 (0.64)^***^
Only from the list of providers	−0.37 (0.10)^***^	−0.88 (0.21)^***^	−1.74 (0.60)^***^
Chronic patients pay			
*Neither deductibles nor co-payments (ref)*			
Both deductibles and co-payments	−0.47 (0.11)^***^	0.02 (0.23)	−0.23 (0.38)
Only co-payments	−0.23 (0.11)^**^	0.21 (0.22)	−1.43 (0.66)^**^
Only deductibles	−0.01 (0.12)	0.03 (0.27)	−1.38 (0.58)^**^
Formal compensation for informal care givers			
*No formal compensation (ref)*			
Informal care compensated	−0.11 (0.10)	1.09 (0.17)^***^	0.09 (0.37)
Informal care compensated, plus access to extra services	0.15 (0.11)	0.95 (0.17)^***^	−4.81 (2.89)^*^
Monthly premium			
*Remained unchanged (ref)*			
Increased	−1.17 (0.12)^***^	−0.69 (0.19)^***^	−1.96 (0.53)^***^
Decreased	−0.47 (0.10)^***^	0.09 (0.18)	−2.43 (0.62)^***^
Constant 1	0.43 (0.10)^***^		
Constant 2	−0.54 (0.17)^***^		
Posterior probability	0.92		
AIC/BIC	9586.9/9975.5		
LL	−4743.4		
N parameters	50		
Number of observations/rows	5’850/17’550		

* Significant at the 10% level.

** Significant at the 5% level.

*** Significant at the 1% level, ‘ref’ indicates the baseline.

Class 1 members were rather cost-concerned (regarding premium increase), and at the same time were in favour of more integrated care structures where care was not managed by one GP only, but rather by a team of professionals. It was shown by their strong preferences of extended access to EMR to all healthcare professionals involved in care (but not all doctors), as well as care coordination performed by an interprofessional team. Gatekeeping, restrictions of access to EMR, and having no designated care coordinator were valued negatively in Class 1 (table 2). Additionally, the members of this class were the most critical towards maintaining cost-sharing for chronic patients, while the attribute of informal care compensation did not seem to influence their choices.

Individuals in class 2 seemed to be the most open to changes in healthcare delivery (table 2). These individuals were most in favour of informal care compensation, had lowest aversion of gatekeeping plans, and were the least responsive to premium changes (only the increase in premium was valued negatively). Other than that, they preferred to have a GP or a team as the care coordinator and extended access to EMR to all professionals or doctors involved in care.

Class 3 respondents had the highest probability of choosing the status quo; they showed reluctance for any attributes diverting from the current situation: e.g. strongest aversion of premium changes both negative and positive, any models with restricted access to the specialists, and informal care compensation. It needs to be noted, that the standard errors in this class were rather high reflecting a high level of randomness in the responses. Additionally, the members of Class 3 preferred the chronic patients being fully exempted from paying the deductibles together with co-payments, and negatively valued only partial exemptions (only co-payments, or only deductibles) (table 2).

#### Class membership description

Compared with Class 1 (majority of respondents), members of Class 2 were younger, higher educated, less wealthy, and were more likely to have low deductibles and gatekeeping plans (table 3). The members of Class 2 were 39% more likely to provide informal care for their relatives. On the other hand, they were 16% more likely to report having one chronic condition and 20% less likely to be multimorbid than the members of Class 1. Noteworthy, the members of Class 2 contained 98% of all individuals who never chose the current situation (status quo option) in the DCE experiment, and related to that, were 12% more likely to express that Swiss healthcare system needed substantial reforms. Additionally, they were 26% more ready to pay higher taxes to improve the healthcare system, and 26% more likely to express that the system when the richest can afford better healthcare than poorest is unjust (table 3).

**Table 3:** Multinomial logistic regression differentiating members of Class 1 (*n* = 419, reference) from members of Class 2 (*n* = 168) and Class 3 (*n* = 290)

	Class 2, 95% OR	Class 3, 95% OR
Socio-demographic characteristics		
Age	0.99***	1.03***
Sex (Female)	0.99	1.03
Income quartile (1)		
2	0.67***	1.05
3	0.87*	0.92
4	0.36***	0.77***
Education (compulsory schooling)		
Apprentice	2.00***	0.67***
High school	1.03	0.56***
College	2.47***	0.76**
University	1.84**	0.74**
Civil status (Single)		
Married	4.16**	1.15**
Divorced	2.37***	0.93
Widowed	3.50***	0.69***
Health-related		
Morbidity (None)		
1	1.16**	0.94
2+	0.80***	0.88***
Has been hospitalized in the past year (yes)	0.62***	0.81***
Insurance-related		
Low deductibles (300 or 500 CHF)	1.16**	1.19***
Gatekeeping model (yes)	1.09*	0.95*
Subsidy for health insurance (yes)	0.68***	1.05
Health literacy		
Confidence in choice of health insurance (not at all confident)	0.55***	0.72***
Low literacy (always experience problems with understanding written medical info)	1.76***	0.98
Informal care		
Provide informal care (yes)	1.39***	0.93*
Receive informal care (yes)	0.44***	0.71***
Opinion and trust in healthcare system in Switzerland		
Healthcare system in Switzerland needs substantial reforms (yes)	1.12**	0.73***
The system when the richest can afford better healthcare than poorest is unfair (yes)	1.26***	1.03
In favour of public fund for obligatory health insurance (yes)	1.10	0.76***
Ready to pay more taxes for better medical care for all in Switzerland (yes)	1.26***	0.98
Never chose current (status quo) model in DCE, N	91 (98%)	0 (0%)
Always chose current (status quo) model in DCE, N (%)	0 (0%)	207 (100%)

* Significant at the 10% level. ** Significant at the 5% level. *** Significant at the 1% level.

The most distinguishing characteristics of membership in Class 3 is that it contained 100% of all those who consistently chose the current situation. Compared with Class 1 (majority), the members of Class 3 were likely the most senior, less wealthy and lower educated (table 3). Moreover, the members of Class 3 were 27% less likely to express that Swiss healthcare system needs large reforms and 24% less likely to be in favour of a public fund introduction for managing obligatory health insurances.

## Discussion

In the present study, we demonstrated that analysing preferences from the perspective of an average individual is not optimal, and various subgroups of the population must be accounted for to develop and communicate healthcare reforms and innovations. We distinguished three distinct classes within our study population: the majority (49%) was open to some reforms mostly for integrated healthcare structures; a smaller class of adults most open to reforms (19%), and a class of conservative individuals, reluctant to changes (32%).

We found that the largest group attached high importance to a stronger role of interprofessional teams in coordinating care, access to the EMR and health insurance premiums. It may indicate awareness of this group on the issues of increasing health expenditures, and lack of coordination among healthcare professionals, which is in line with earlier surveys highlighting coordination gaps in Swiss healthcare.^27–29^ Specifically, in case of healthcare delivery policy-making, it is important to develop communication strategy and information campaigns reflecting the needs and interests of this group and emphasize benefits from better information exchange among the providers with possible access to electronic health records, the leading roles of coordinating healthcare professionals and measures to prevent premium increases. The findings that the largest population class prefers to switch away from the care centred around one general practitioner towards integrated care seem promising, as it coincides with the general agenda of Swiss policy.^6^^,^^30^ There is the significant scope to broaden the uptake of non-physician professionals in primary care in Switzerland, whereby the inclusion of new professional roles can lead to higher care quality and satisfaction, without affecting costs.^31^

Secondly, we demonstrated that there is a group of most open to reforms individuals, who are younger in the adult population, healthier, express an opinion that the system where the existing healthcare system is unjust, and they are ready to pay more taxes for more-equity healthcare. Similar findings were observed in the USA, where respondents who felt that income-based healthcare inequalities were unfair, were more likely to support major health system reform, although much larger health and income disparities were found in the USA than in Switzerland.^32^ In an earlier study from Switzerland, it was found that the majority of the respondents were in favour of broad public-health reforms,^33^ whereby those interested in politics expressed more support towards healthcare access-improving reforms. This population subgroup is more likely to actively participate in the political discussion in healthcare, and consequently, it would be beneficial to involve them into discussions about the upcoming reforms to reach greater patient involvement.

Finally, the last group of respondents were most senior, less wealthy, and less educated yet more conservative individuals, usually choosing the status quo and showed reluctance for any attributes diverting from the current situation, especially limiting the freedom of provider choices. Individuals in this group expressed an opinion that Switzerland needs little or no healthcare reforms and were less likely to support any alternative means of financing healthcare (e.g. single fund or tax-based). Individuals of older age in Switzerland generally showed higher satisfaction with current situation in healthcare quality and healthcare policy, compared with younger adults^16^^,^^18^^,^^34^ and to the other countries.^35^ Moreover, earlier studies from Switzerland showed that due to economic reasons,^18^^,^^20^^,^^36^ the more senior and less healthy individuals were less likely to give up their current healthcare models. However, it is not clear whether the reluctance to any changes is related to general satisfaction with system performance, or the lack of up-to-date information about the current healthcare issues the country is facing. This would be the population subgroup benefitting most from the nation-wide communication strategies, focusing on the reasons for changes as the existing situation is suboptimal, and potentially healthcare education campaigns.

Our study makes an important contribution to the literature exploring preferences among Swiss population groups towards potential healthcare system changes to strengthen policy decisions. Although earlier studies and the history of the Swiss votes in healthcare showed strong attachment of the Swiss population to status quo,^2^^,^^3^^,^^18–20^ we were able to demonstrate that not all individuals are reluctant to changes. Current study’s novelty is in demonstrating the preferences, shares and characteristics of population subgroups most open to healthcare reforms, accepting only specific reforms and those who are unwilling to accept changes. In the Swiss context, these findings are crucial to develop the tailored reforms and establish the effective communication strategy focused on the needs of specific population groups. Currently, as the alternative models of healthcare delivery for chronic patients are under heated debates, the results of our study would be of high relevance for policymakers, public-health professionals, health insurance representatives, researchers developing innovative policy plans, and engaged professionals in the discussion of potential adoption of healthcare reforms. We reached a relatively large sample of Swiss residents aged 50+ and reduced the selection bias occurring in the online sampling studies by applying dual age-adapted data collection mode.^8^ Moreover, we involved multiple stakeholders together with the general population and patients into the development of the DCE study, from the very first stages resulting in potentially higher credibility and acceptability of the study results.^34^ Finally, the engagement of multiple actors allowed defining care delivery characteristics, previously not mentioned in the literature (e.g. informal care compensation or exemption of chronic patients from payments).

However, our study is subject to limitations. First, our sample consisted of an adult population residing in only Swiss French-speaking regions, not representative of the general Swiss population. There are likely cultural differences in perceptions of healthcare in various language regions in Switzerland,^36^^,^^37^ therefore, investigating language-specific preferences towards new models of healthcare may be of interest in the future research. We focused on individuals aged 50+ as they were the most likely to benefit from innovative better-coordinated healthcare models, and were characterized by particularly pronounced attachments to status quo.^18^ Second, specific DCE methodological limitations need to be mentioned, such as concerns of external validity due to hypothetical nature of choices and its consistency with real-world situations, simplifying heuristics of respondents, and insufficient respondents’ involvement into the tasks limiting the quality of the data. We tried to minimize such occurrences by testing the survey during multiple steps during focus groups and an online pre-test. Third, earlier studies revealed the concerns about the growing complexity of the DCE models, which not necessarily adds to the precision and robustness of conclusions^21^ but rather risk overfitting data.^38^ We used a relatively accessible latent class analysis (LCA) model and limited the number of classes to an interpretable amount, so we were able to distinguish them well enough to answer the research question.

To conclude, for ensuring potential acceptability of the healthcare reform or innovation, it is key to understand the population preferences, accounting for differences among distinct groups. We distinguished the population profiles generally more open to reforms, those open to limited specific changes, and those preferring no changes at all. The development of the potential reforms and the subsequent communication approach should be implemented stepwise with various intensity and focus of changes, tailored to the preferences of several major groups: e.g. better-coordinated care structures, more cost containment strategies, better integration of informal care, promotion of extended access to the EMR for health professionals.

## Supplementary Material

ckae004_Supplementary_Data

## Data Availability

The dataset is available from the authors on request. Key points We distinguished three distinct classes: the majority open to some reforms (e.g. integrated healthcare), progressive younger adults open to major reforms, and senior conservative individuals attached to status quo. We were able to demonstrate that not all Swiss adults aged 50+ were reluctant to changes. Our approach helped to foresee what population groups were more likely to accept healthcare reforms, and what type of reforms were of largest value to these population groups.
