# Supplementary material for: Preferences for innovations in healthcare delivery models in the Swiss elderly population: a latent class, choice modelling study
Source: Eur J Public Health. 2024 Jan 18;34(2):260–6. doi: 10.1093/eurpub/ckae004 (PMC10990495; doi:10.1093/eurpub/ckae004)
Supplement: ckae004_Supplementary_Data [file ckae004_supplementary_data.zip › ckae004_Supplementary_Data/ejph-2023-06-om-0339-File002.docx]

# Appendix

**Table 1.** Number of classes optimization based on AIC and BIC in the latent class model.

| Classes | AIC | BIC |
| --- | --- | --- |
| 2 | 9945.7 | 9912.7 |
| 3 | 9881.0 | 9831.0 |
| 4 | 9915.3 | 9848.3 |
| 5 | 9924.5 | 9840.5 |
| 6 | 9984.5 | 9883.4 |
| 7 | 10118.3 | 10000.3 |
