# Supplementary material for: Preferences for innovations in healthcare delivery models in the Swiss elderly population: a latent class, choice modelling study
Source: Eur J Public Health. 2024 Jan 18;34(2):260–6. doi: 10.1093/eurpub/ckae004 (PMC10990495; doi:10.1093/eurpub/ckae004)
Supplement: ckae004_Supplementary_Data [file ckae004_supplementary_data.zip › ckae004_Supplementary_Data/ejph-2023-06-om-0339-File003.pdf]

unisanté

Centre universitaire de médecine générale
et santé publique · Lausanne

Étude sur le système de soins en Suisse

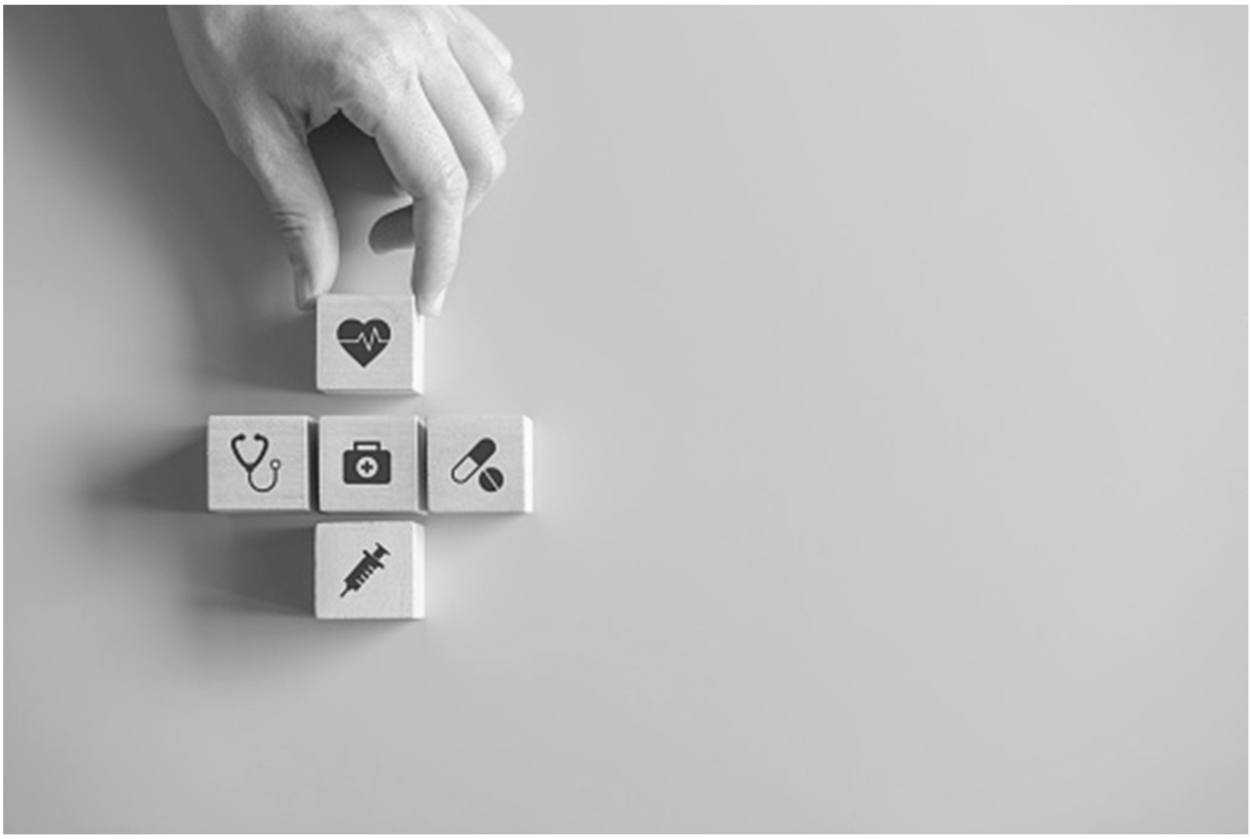

Questionnaire papier
2021

V1XXX

INSTRUCTIONS POUR COMPLETER LE QUESTIONNAIRE

La présente enquête porte sur les préférences par rapport au système de santé auprès de la population suisse âgée de 50 ans et plus.

Il faut compter environ 25 minutes pour répondre au questionnaire. Vos données et réponses seront traitées de manière **confidentielle et anonyme**.

Nous vous remercions chaleureusement de prendre le temps de répondre à notre enquête.

Voici quelques brèves instructions et précisions :

- Veuillez utiliser un **stylo à bille foncé** ou un autre stylo bien lisible pour remplir le formulaire.
- Veuillez entrer votre réponse avec une croix dans le champ de votre choix : ⊗
- Si vous avez coché une case incorrecte, veuillez biffer la case erronée en dépassant clairement les bords et faire une nouvelle croix dans la case souhaitée : ~~⊗~~ ⊗
- Veuillez ne donner **qu'une seule réponse** par question, sauf pour les questions incluant la remarque « Plusieurs réponses possibles ». Ces dernières sont identifiables par des cases carrées. ☒
- Le symbole ✍ ___ indique une réponse écrite et sans abréviation.

Si vous avez des questions, n'hésitez pas à nous contacter :

- Téléphone : **021 692 46 72**
- Courriel : **sante2021@fors.unil.ch**

Merci de nous retourner le questionnaire rempli en utilisant l'enveloppe retour préaffranchie jointe à notre courrier :

FORS - 5005
Université de Lausanne
Bâtiment Géopolis
1015 Lausanne

SECTION 1 – QUESTIONS GÉNÉRALES

Q1 Vous êtes ?

- ○ 1 Une femme
- ○ 2 Un homme
- ○ 3 Autre/non-binaire

Q2 Quelle est votre année de naissance ?

✍ |_|_|_|_|

Q3 Quel est votre canton de résidence en Suisse ?

- ○ 1 Berne (région du Jura bernois)
- ○ 2 Fribourg
- ○ 3 Genève
- ○ 4 Jura
- ○ 5 Neuchâtel
- ○ 6 Valais (Bas-Valais)
- ○ 7 Vaud
- ○ 8 Autre

Q4 Quel est votre état civil ?

- ○ 1 Célibataire
- ○ 2 Marié·e / Partenariat enregistré
- ○ 3 Séparé·e / Divorcé·e / Partenariat dissous
- ○ 4 Veuf/veuve

Q5 Laquelle des propositions suivantes décrit le mieux votre situation actuelle ?

- ○ 1 Vit seul·e
- ○ 2 Famille monoparentale (un parent avec enfant(s))
- ○ 3 En couple sans enfant
- ○ 4 En couple avec enfant(s)
- ○ 5 Vit en institution (EMS)
- ○ 6 Autre (veuillez préciser) ✍ ____________

Q6 Quelle est la formation la plus élevée que vous avez achevée ?

- ○ 1 Aucune formation scolaire achevée
- ○ 2 École obligatoire (école primaire, cycle d'orientation)
- ○ 3 Apprentissage, formation professionnelle
- ○ 4 École post-obligatoire (p. ex. collège, école de commerce, école de culture générale)
- ○ 5 Éducation tertiaire (p. ex. université, haute école)

Q7 Quelle option décrit le mieux votre situation professionnelle actuelle ?

- ○1 Employé·e à temps plein (80-100%)
- ○2 Employé·e à temps partiel (moins de 80%)
- ○3 Travailleur/travailleuse indépendant·e
- ○4 Sans emploi
- ○5 Retraité·e
- ○6 À l'assurance-invalidité (AI)
- ○7 À la maison pour faire les tâches domestiques et familiales
- ○8 Autre (veuillez préciser) ✍

Q8 Quelle est la catégorie professionnelle qui correspond le mieux à l'activité que vous exercez (ou que vous exerciez) ?

- ○1 Manœuvre, ouvrier/ouvrière
- ○2 Ouvrier/ouvrière qualifié·e, contremaître
- ○3 Agriculteur/agricultrice
- ○4 Employé·e sans formation (par exemple : aide de bureau)
- ○5 Employé·e qualifié·e (par exemple : secrétaire, comptable)
- ○6 Cadre moyen·ne (par exemple : technicien·ne, enseignant·e)
- ○7 Indépendant·e du petit commerce, artisan·e
- ○8 Cadre supérieur·e (par exemple : économiste, juriste dans une entreprise)
- ○9 Profession libérale (par exemple : médecin, juriste)
- ○10 Directeur/directrice, chef/cheffe d'entreprise ou de service public
- ○11 Je n'ai jamais exercé d'activité professionnelle
- ○12 Autre (veuillez préciser) ✍

Q9 Si vous additionnez toutes les sources de revenus, quel est le revenu net total de votre ménage par mois ?

- ○1 Moins de 3'000 CHF
- ○2 Entre 3'000 CHF et 5'000 CHF
- ○3 Entre 5'001 CHF et 7'000 CHF
- ○4 Entre 7'001 CHF et 9'000 CHF
- ○5 Entre 9'001 CHF et 11'000 CHF
- ○6 Entre 11'001 CHF et 13'000 CHF
- ○7 Plus de 13'000 CHF
- ○8 Je ne sais pas
- ○9 Je ne souhaite pas répondre

SECTION 2 – PRÉFÉRENCES CONCERNANT LE SYSTÈME DE SANTÉ

Dans cette section, nous souhaitons mieux comprendre vos préférences concernant le système de santé en suisse et identifier certaines caractéristiques particulièrement importantes à vos yeux.

Pour ce faire, nous allons vous demander de faire une série de choix pour 8 scénarios proposant deux options décrivant l'accès et l'utilisation des soins en Suisse.

Ces 8 scénarios sont différents même s'ils se ressemblent. La répétition de ces scénarios nous aide à comprendre l'importance que vous accordez aux différentes caractéristiques du système de santé.

Chaque option sera décrite à travers 6 caractéristiques qui vous seront expliquées ci-après.

Veuillez porter une attention particulière aux six encadrés suivants, car ils sont extrêmement importants pour la compréhension de la suite de l'enquête.

Notez que certaines des caractéristiques ne reflètent pas la réalité suisse, mais décrivent des politiques qui pourraient être mises en place dans le futur.

DESCRIPTION DES 6 CARACTÉRISTIQUES PRÉSENTÉES DANS LES SCÉNARIOS : 1/6

Caractéristique 1 : Quel·le(s) professionnel·le(s) a accès à mon dossier électronique du patient ?

Explication : Le dossier électronique du patient (DEP) est un dossier informatisé sécurisé qui permet de regrouper différentes informations concernant votre santé. Depuis 2017, une loi fédérale oblige les hôpitaux et les maisons de retraite à adopter un DEP ; ce dernier est facultatif pour les soins ambulatoires. Son accès est garanti en tout temps et en tout lieu via une connexion internet sécurisée ; le traitement des informations qu'il contient garantit la confidentialité de vos données.

Cette caractéristique peut prendre les modalités suivantes :

A. **Mon/ma médecin de famille** uniquement
B. **Tou·te·s les médecins impliqué·e·s** dans ma prise en charge
C. **Tou·te·s les professionnel·le·s de santé** (médecins et non médecins) **impliqué·e·s** dans ma prise en charge
D. **Tou·te·s les professionnel·le·s de santé impliqué·e·s** dans ma prise en charge ainsi que **ma caisse d'assurance maladie de base**

DESCRIPTION DES 6 CARACTÉRISTIQUES PRÉSENTÉES DANS LES SCÉNARIOS : 2/6

Caractéristique 2 : Quelle est la variation de ma prime mensuelle d'assurance maladie de base ?

Explication : Il s'agit de la cotisation mensuelle que vous devez verser à votre caisse d'assurance maladie de base.

Cette caractéristique peut prendre les modalités suivantes :

A. Ma prime mensuelle diminue de 50 francs (**- 50 CHF**)
B. Ma prime mensuelle diminue de 100 francs (**- 100 CHF**)
C. Ma prime mensuelle **reste la même**
D. Ma prime mensuelle augmente de 50 francs (**+ 50 CHF**)
E. Ma prime mensuelle augmente de 100 francs (**+ 100 CHF**)

DESCRIPTION DES 6 CARACTÉRISTIQUES PRÉSENTÉES DANS LES SCÉNARIOS : 3/6

Caractéristique 3 : Quel·le(s) professionnel·le(s) est(sont) clairement désigné·e(s) pour assurer la coordination de mes soins ?

<u>Explication</u> : Une coordination des soins est nécessaire dès que plusieurs professionnel·le·s de santé sont impliqué·e·s dans vos soins. Elle nécessite la communication d'informations entre vous (le patient) et les professionnels en y associant vos proches. Outre la communication de l'information, un·e professionnel·le désigné·e pour coordonner vos soins aura les tâches suivantes : surveiller et coordonner votre plan de traitement, fournir des informations sur votre état de santé, répondre à vos questions, définir avec vous un plan de soins ou de santé pour répondre à vos besoins personnels, consulter et collaborer avec d'autres services de santé, prestataires de soins et spécialistes.

<u>Cette caractéristique peut prendre les modalités suivantes :</u>

A. **Aucun·e professionnel·le de santé** n'est clairement désigné·e
B. **Mon/ma médecin de famille**
C. **Un·e professionnel·le de santé** non médecin (ex. infirmier/infirmière, assistant·e médicale)
D. **Une équipe de soins** incluant plusieurs professionnel·le·s de santé (médecins et non médecins)
E. Un·e référent·e (médecin ou non médecin) de **ma caisse d'assurance maladie de base**

DESCRIPTION DES 6 CARACTÉRISTIQUES PRÉSENTÉES DANS LES SCÉNARIOS : 4/6

Caractéristique 4 : Comment ai-je accès aux médecins spécialistes ?

<u>Explication</u> : Aujourd'hui, dans le modèle standard d'assurance maladie de base, il n'y a aucune restriction quant au choix de vos médecins et vous pouvez vous adresser directement à un·e médecin spécialiste. Avec certains modèles alternatifs d'assurance vous devez passer par votre médecin de famille afin qu'il/elle vous adresse à un·e médecin spécialiste (à l'exception des consultations d'urgence et des consultations chez un·e ophtalmologue ou un·e gynécologue).

<u>Cette caractéristique peut prendre les modalités suivantes :</u>

A. Possibilité de **consulter directement** un·e spécialiste
B. Pas de possibilité de consulter directement un·e spécialiste ; c'est **mon/ma médecin de famille** qui m'oriente vers un·e spécialiste
C. Je peux consulter **directement uniquement les spécialistes** indiqué·e·s dans **une liste définie** dans mon contrat d'assurance maladie de base

DESCRIPTION DES 6 CARACTÉRISTIQUES PRÉSENTÉES DANS LES SCÉNARIOS : 5/6

Caractéristique 5 : Les assuré·e·s atteint·e·s d'une maladie chronique paient-ils/elles une franchise et/ou une quote-part ?

Explication : Actuellement, dans le cadre de l'assurance maladie de base, les assuré·e·s, en plus de leur prime mensuelle, doivent payer les premiers frais de santé qu'on appelle franchise (montant allant de 300 CHF à 2'500 CHF pour les adultes). Une fois la franchise atteinte, l'assuré·e paie une quote-part (i.e. une participation aux frais) de 10% pour un montant maximum de 700 CHF par an.

Cette caractéristique peut prendre les modalités suivantes :

A. Les assuré·e·s atteint·e·s d'une maladie chronique payent **une franchise et une quote-part**
B. Les assuré·e·s atteint·e·s d'une maladie chronique ne payent **que la quote-part**
C. Les assuré·e·s atteint·e·s d'une maladie chronique ne payent **que la franchise**
D. Les assuré·e·s atteint·e·s d'une maladie chronique ne payent **ni franchise ni quote-part**

DESCRIPTION DES 6 CARACTÉRISTIQUES PRÉSENTÉES DANS LES SCÉNARIOS : 6/6

Caractéristique 6 : Les soins et le soutien aux proches aidant·e·s sont-ils compensés ?

Explication : Actuellement, les soins et le soutien, apportés par des proches aidant·e·s, c'est-à-dire des personnes s'occupant de leurs proches nécessitant une assistance, ponctuelle ou permanente, ne sont pas compensés formellement.

Cette caractéristique peut prendre les modalités suivantes :

A. Les soins et le soutien sont **formellement compensés** (ex : réduction de prime, congés payés supplémentaires)
B. Les soins et le soutien sont **formellement compensés** et les proches aidant·e·s ont accès à des **services spécifiques** (ex : information, formation, soutien psychologique)
C. Les soins et le soutien ne sont **pas formellement compensés**

Pour chacune des 8 questions suivantes, nous vous demandons de **choisir l'option que vous préférez** entre les deux présentées.

Lorsque vous faites ce choix :

- Considérez attentivement l'ensemble des six caractéristiques
- Deux caractéristiques sur six ne diffèrent pas entre les options (marquées en gris foncé)
- Considérez que toutes les autres caractéristiques du système de santé (non présentées dans le tableau) sont les mêmes entre les deux options
- Répondez à tous les choix, en considérant à chaque fois que seules ces deux options sont disponibles
- Il n'y a pas de bonne ou de mauvaise réponse !

À la page suivante, nous vous montrons **un exemple** d'un tel scénario.

Parmi les options suivantes, laquelle préférez-vous ?

	**Option 1**	**Option 2**
**Quel·le(s) professionnel·e(s) a accès à mon dossier électronique du patient ?**	Mon/ma médecin de famille uniquement	Tou·te·s les professionnel·le·s de santé impliqué·e·s ainsi que ma caisse d'assurance maladie de base
**Qui coordonne mes soins ?**	Une équipe de soins	Aucun·e professionnel·le de santé
**Accès aux spécialistes**	**Consultation directe possible (libre choix)**	**Consultation directe possible (libre choix)**
**Que paient les assuré·e·s atteint·e·s de maladie chronique ?**	Ni franchise, ni quote-part	Uniquement la quote-part
**Compensation formelle des soins et du soutien aux proches aidant·e·s**	Oui	Non
**Variation de ma prime mensuelle de mon assurance maladie de base**	**+ 50 CHF**	**+ 50 CHF**

	**Option 1**	**Option 2**
Votre choix :	○ 1	⊗ 2

	**Votre situation actuelle**	**L'option sélectionnée ci-dessus**
Si vous pouviez maintenant garder votre situation actuelle : que choisiriez-vous ?	○ 1	⊗ 2

Dans cet exemple, la personne préfère l'option 2 à l'option 1 et à la situation actuelle.

À partir de maintenant, nous vous demanderons de faire une série de choix similaires à l'exemple précédent.

Si besoin, vous pouvez relire les définitions des caractéristiques (présentées aux pages 5 à 7) lorsque vous répondez aux différents scénarios.

Scénario 1 : Parmi les options suivantes, laquelle préférez-vous ?

	Option 1	Option 2
**Quel·le(s) professionnel·e(s) a accès à mon dossier électronique du patient ?**	Mon/ma médecin de famille uniquement	Tou·te·s les professionnel·le·s de santé impliqué·e·s ainsi que ma caisse d'assurance maladie de base
**Qui coordonne mes soins ?**	Une équipe de soins	Aucun·e professionnel·le de santé
**Accès aux spécialistes**	**Consultation directe possible (libre choix)**	**Consultation directe possible (libre choix)**
**Que paient les assuré·e·s atteint·e·s de maladie chronique ?**	Ni franchise, ni quote-part	Uniquement la quote-part
**Compensation formelle des soins et du soutien aux proches aidant·e·s**	Oui	Non
**Variation de ma prime mensuelle de mon assurance maladie de base**	**+50 CHF**	**+50 CHF**

	**Option 1**	**Option 2**
Votre choix :	○ 1	○ 2

	**Votre situation actuelle**	**L'option sélectionnée ci-dessus**
Si vous pouviez maintenant garder votre situation actuelle : que choisiriez-vous ?	○ 1	○ 2

Scénario 2 : Parmi les options suivantes, laquelle préférez-vous ?

	Option 1	Option 2
**Quel·le(s) professionnel·e(s) a accès à mon dossier électronique du patient ?**	**Mon/ma médecin de famille uniquement**	**Mon/ma médecin de famille uniquement**
**Qui coordonne mes soins ?**	Mon/ma médecin de famille	Un·e référent·e de ma caisse d'assurance maladie de base
**Accès aux spécialistes**	Consultation directe possible si le/la médecin figure dans une liste (choix restreint)	Nécessité d'être orienté·e par le/la médecin de famille (gatekeeping)
**Que paient les assuré·e·s atteint·e·s de maladie chronique ?**	Uniquement la quote-part	Ni franchise, ni quote-part
**Compensation formelle des soins et du soutien aux proches aidant·e·s**	Non	Oui, et accès à des services de soutien spécifiques
**Variation de ma prime mensuelle de mon assurance maladie de base**	**-50 CHF**	**-50 CHF**

	**Option 1**	**Option 2**
Votre choix :	○ 1	○ 2

	**Votre situation actuelle**	**L'option sélectionnée ci-dessus**
Si vous pouviez maintenant garder votre situation actuelle : que choisiriez-vous ?	○ 1	○ 2

Scénario 3 : Parmi les options suivantes, laquelle préférez-vous ?

	Option 1	Option 2
**Quel·le(s) professionnel·e(s) a accès à mon dossier électronique du patient ?**	**Tous les professionnels de santé impliqués ainsi que ma caisse d'assurance maladie de base**	**Tous les professionnels de santé impliqués ainsi que ma caisse d'assurance maladie de base**
**Qui coordonne mes soins ?**	Mon médecin de famille	Un professionel de santé non médecin
**Accès aux spécialistes**	Consultation directe possible si le médecin figure dans une liste (choix restreint)	Consultation directe possible (libre choix)
**Que paient les assuré·e·s atteint·e·s de maladie chronique ?**	Uniquement la quote-part	Une franchise et une quote-part
**Compensation formelle des soins et du soutien aux proches aidant·e·s**	Oui, et accès à des services de soutien spécifiques	Non
**Variation de ma prime mensuelle de mon assurance maladie de base**	**-100 CHF**	**-100 CHF**

	**Option 1**	**Option 2**
Votre choix :	○ 1	○ 2

	**Votre situation actuelle**	**L'option sélectionnée ci-dessus**
Si vous pouviez maintenant garder votre situation actuelle : que choisiriez-vous ?	○ 1	○ 2

Scénario 4 : Parmi les options suivantes, laquelle préférez-vous ?

	Option 1	Option 2
**Quel·le(s) professionnel·e(s) a accès à mon dossier électronique du patient ?**	Mon/ma médecin de famille uniquement	Tou·te·s les professionnel·le·s de santé impliqué·e·s ainsi que ma caisse d'assurance maladie de base
**Qui coordonne mes soins ?**	Aucun·e professionnel·le de santé	Un·e professionnel·le de santé non médecin
**Accès aux spécialistes**	Consultation directe possible (libre choix)	Nécessité d'être orienté·e par le/la médecin de famille (gatekeeping)
**Que paient les assuré·e·s atteint·e·s de maladie chronique ?**	**Uniquement la quote-part**	**Uniquement la quote-part**
**Compensation formelle des soins et du soutien aux proches aidant·e·s**	Non	Oui, et accès à des services de soutien spécifiques
**Variation de ma prime mensuelle de mon assurance maladie de base**	**+100 CHF**	**+100 CHF**

	**Option 1**	**Option 2**
Votre choix :	○ 1	○ 2

	**Votre situation actuelle**	**L'option sélectionnée ci-dessus**
Si vous pouviez maintenant garder votre situation actuelle : que choisiriez-vous ?	○ 1	○ 2

Scénario 5 : Parmi les options suivantes, laquelle préférez-vous ?

	**Option 1**	**Option 2**
**Quel·le(s) professionnel·e(s) a accès à mon dossier électronique du patient ?**	Mon médecin de famille uniquement	Tous les médecins impliqués dans ma prise en charge
**Qui coordonne mes soins ?**	Une équipe de soins	Un professionel de santé non médecin
**Accès aux spécialistes**	Consultation directe possible (libre choix)	Consultation directe possible si le médecin figure dans une liste (choix restreint)
**Que paient les assuré·e·s atteint·e·s de maladie chronique ?**	**Une franchise et une quote-part**	**Une franchise et une quote-part**
**Compensation formelle des soins et du soutien aux proches aidant·e·s**	**Non**	**Non**
**Variation de ma prime mensuelle de mon assurance maladie de base**	-50 CHF	-100 CHF

	**Option 1**	**Option 2**
Votre choix :	○ 1	○ 2

	**Votre situation actuelle**	**L'option sélectionnée ci-dessus**
Si vous pouviez maintenant garder votre situation actuelle : que choisiriez-vous ?	○ 1	○ 2

Scénario 6 : Parmi les options suivantes, laquelle préférez-vous ?

	Option 1	Option 2
**Quel·le(s) professionnel·e(s) a accès à mon dossier électronique du patient ?**	Tou·te·s les médecins impliqué·e·s dans ma prise en charge	Tou·te·s les professionnel·le·s de santé impliqué·e·s ainsi que ma caisse d'assurance maladie de base
**Qui coordonne mes soins ?**	Mon/ma médecin de famille	Une équipe de soins
**Accès aux spécialistes**	**Nécessité d'être orienté·e par le/la médecin de famille (gatekeeping)**	**Nécessité d'être orienté·e par le/la médecin de famille (gatekeeping)**
**Que paient les assuré·e·s atteint·e·s de maladie chronique ?**	Uniquement la franchise	Uniquement la quote-part
**Compensation formelle des soins et du soutien aux proches aidant·e·s**	**Non**	**Non**
**Variation de ma prime mensuelle de mon assurance maladie de base**	+50 CHF	+100 CHF

	**Option 1**	**Option 2**
Votre choix :	○ 1	○ 2

	**Votre situation actuelle**	**L'option sélectionnée ci-dessus**
Si vous pouviez maintenant garder votre situation actuelle : que choisiriez-vous ?	○ 1	○ 2

Scénario 7 : Parmi les options suivantes, laquelle préférez-vous ?

	**Option 1**	**Option 2**
**Quel·le(s) professionnel·e(s) a accès à mon dossier électronique du patient ?**	Tous les médecins impliqués dans ma prise en charge	Tous les professionnels de santé impliqués dans ma prise en charge
**Qui coordonne mes soins ?**	Une équipe de soins	Mon médecin de famille
**Accès aux spécialistes**	**Nécessité d'être orienté·e par le médecin de famille (gatekeeping)**	**Nécessité d'être orienté·e par le médecin de famille (gatekeeping)**
**Que paient les assuré·e·s atteint·e·s de maladie chronique ?**	Uniquement la franchise	Une franchise et une quote-part
**Compensation formelle des soins et du soutien aux proches aidant·e·s**	**Oui, et accès à des services de soutien spécifiques**	**Oui, et accès à des services de soutien spécifiques**
**Variation de ma prime mensuelle de mon assurance maladie de base**	-100 CHF	+50 CHF

	**Option 1**	**Option 2**
Votre choix :	○ 1	○ 2

	**Votre situation actuelle**	**L'option sélectionnée ci-dessus**
Si vous pouviez maintenant garder votre situation actuelle : que choisiriez-vous ?	○ 1	○ 2

Scénario 8 : Parmi les options suivantes, laquelle préférez-vous ?

	Option 1	Option 2
**Quel·le(s) professionnel·e(s) a accès à mon dossier électronique du patient ?**	Mon/ma médecin de famille uniquement	Tou·te·s les médecins impliqué·e·s dans ma prise en charge
**Qui coordonne mes soins ?**	Une équipe de soins	Un·e professionnel·le de santé non médecin
**Accès aux spécialistes**	Consultation directe possible (libre choix)	Consultation directe possible si le/la médecin figure dans une liste (choix restreint)
**Que paient les assuré·e·s atteint·e·s de maladie chronique ?**	**Une franchise et une quote-part**	**Une franchise et une quote-part**
**Compensation formelle des soins et du soutien aux proches aidant·e·s**	**Non**	**Non**
**Variation de ma prime mensuelle de mon assurance maladie de base**	-50 CHF	-100 CHF

	**Option 1**	**Option 2**
Votre choix :	○ 1	○ 2

	**Votre situation actuelle**	**L'option sélectionnée ci-dessus**
Si vous pouviez maintenant garder votre situation actuelle : que choisiriez-vous ?	○ 1	○ 2

SECTION 3 – SITUATION MÉDICALE ET UTILISATION DU SYSTÈME DE SANTÉ

Nous aimerions maintenant vous poser quelques questions sur votre situation médicale et votre utilisation du système de santé.

Q10 Comment qualifieriez-vous votre état de santé en général ?

- ○ 1 Très bon
- ○ 2 Bon
- ○ 3 Ni bon, ni mauvais
- ○ 4 Mauvais
- ○ 5 Très mauvais

Q11 Au cours des 12 derniers mois, avez-vous été en traitement, ou avez-vous souffert des problèmes de santé suivants, diagnostiqués par un·e médecin ?
Cocher tout ce qui vous concerne, plusieurs réponses possibles

- ☐ 1 Tension artérielle trop élevée (hypertension)
- ☐ 2 Cholestérol élevé dans le sang (graisses)
- ☐ 3 Angine de poitrine ou angor, infarctus du myocarde, crise cardiaque (problème coronarien)
- ☐ 4 Insuffisance cardiaque, maladie des valves ou du muscle cardiaque
- ☐ 5 Attaque cérébrale (ictus, accident vasculaire cérébral-AVC)
- ☐ 6 Diabète (type I ou type II)
- ☐ 7 Maladie pulmonaire chronique (asthme, bronchite chronique, BPCO (broncho-pneumopathie chronique), emphysème)
- ☐ 8 Ostéoporose
- ☐ 9 Arthrose ou arthrite
- ☐ 10 Cancer, tumeur maligne, lymphome
- ☐ 11 Ulcère de l’estomac, du duodénum, ulcère peptique
- ☐ 12 Maladie inflammatoire chronique de l’intestin (maladie de Crohn, colite ulcéreuse)
- ☐ 13 Dépression
- ☐ 14 Maladie de Parkinson, maladie d’Alzheimer
- ☐ 15 Infection par le VIH
- ☐ 16 Covid-19
- ☐ 17 Aucun de ces problèmes de santé

Q12 Quel modèle d'assurance-maladie avez-vous personnellement pour l'assurance de base obligatoire ?

- ○ 1 Modèle d'assurance ordinaire
- ○ 2 Modèle d'assurance en réseau de santé (HMO)
- ○ 3 Modèle du médecin de famille
- ○ 4 Modèle prévoyant une consultation médicale par téléphone au préalable / avant chaque visite médicale
- ○ 5 Autre (veuillez préciser) ✍
- ○ 6 Je ne sais pas

Q13 Quelle est votre franchise annuelle personnelle ?
La franchise est la part de vos frais médicaux qui est à votre charge (non prise en compte par l'assurance).

- ○ 1 300 CHF
- ○ 2 500 CHF
- ○ 3 1'000 CHF
- ○ 4 1'500 CHF
- ○ 5 2'000 CHF
- ○ 6 2'500 CHF
- ○ 7 Je ne sais pas

Q14 Quel est le montant de votre prime mensuelle pour l'assurance de base ?
(sans prendre en compte l'aide de l'Etat / subside si vous en bénéficiez)

- ○ 1 Moins de 200 CHF
- ○ 2 200 - 250 CHF
- ○ 3 251 - 300 CHF
- ○ 4 301 - 350 CHF
- ○ 5 351 - 400 CHF
- ○ 6 401 - 450 CHF
- ○ 7 451 - 500 CHF
- ○ 8 501 - 550 CHF
- ○ 9 551 - 600 CHF
- ○ 10 Plus de 600 CHF
- ○ 11 Je ne sais pas

Q15 Recevez-vous actuellement des subventions du canton pour vos primes d'assurance-maladie ?

- ○ 1 Oui
- ○ 2 Non
- ○ 3 Je ne sais pas

Q16 Avez-vous souscrit à au moins une assurance complémentaire (ex : médecines complémentaires, soins dentaires, hospitalisation, lunettes, couverture à l'étranger) ?

- ○ 1 Oui
- ○ 2 Non
- ○ 3 Je ne sais pas

Q17 Lorsqu'il s'agit de choisir un contrat d'assurance maladie de base, êtes-vous confiant·e en vos capacités à sélectionner celui qui vous convient le mieux ?

- ○ 1 Très confiant·e
- ○ 2 Plutôt confiant·e
- ○ 3 Ni confiant·e, ni pas confiant·e
- ○ 4 Plutôt pas confiant·e
- ○ 5 Pas du tout confiant·e

Q18 Au cours des 12 derniers mois, vous est-il déjà arrivé de renoncer, pour des raisons financières, à certains soins de santé ?

- ○ 1 Oui
- ○ 2 Non

Q19 Avez-vous un·e médecin de famille ou un·e médecin généraliste personnel·le ?
Le/la médecin de famille est le/la médecin chez lequel/laquelle vous pouvez vous rendre pour la plupart de vos problèmes de santé.

- ○ 1 Oui

  ↳ **Au cours des 12 derniers mois, au total combien de fois vous êtes-vous rendu·e chez votre médecin de famille, ou un·e autre médecin généraliste ?**

  Nombre de fois (en chiffres) ✍ ______

- ○ 2 Non
- ○ 3 Je ne sais pas

Q19 bis **Au cours des 12 derniers mois, avez-vous consulté un·e médecin spécialiste *(y compris gynécologue et ophtalmologue mais sans compter les médecins dentistes)* ?**

○ 1 Oui

↳ **Au cours des 12 derniers mois, au total combien de fois vous êtes-vous rendu·e chez un spécialiste *(y compris gynécologue et ophtalmologue mais sans compter les médecins dentistes)* ?**

Nombre de fois (en chiffres) ✍ ______

○ 2 Non

Q20 **Au cours des 12 derniers mois, avez-vous été hospitalisé·e ?**
Prenez-en compte les séjours en service de médecine, de chirurgie, de psychiatrie ou tout autre service spécialisé.

○ 1 Oui

↳ **Au total, combien de nuits avez-vous passées à l'hôpital au cours des 12 derniers mois ?**

Nombre de nuits (en chiffres) ✍ ______

○ 2 Non

Q21 **Au cours des 12 derniers mois, avez-vous consulté un service d'urgences hospitalier directement, sans y avoir été envoyé·e par un médecin ou une ambulance ?**

○ 1 Oui

↳ **Combien de fois avez-vous consulté un service d'urgences hospitalier directement ?**

Nombre de fois (en chiffres) ✍ ______

○ 2 Non

Q22 **Au cours des 12 derniers mois, avez-vous passé une ou plusieurs nuits dans un home médicalisé (type EMS) ?**

○ 1 Oui

○ 2 Non

Q23 Au cours des 12 derniers mois, avez-vous, pour des raisons de santé, reçu de l'aide pour vos commissions, vos repas, vos soins, ou vos tâches administratives ?

- ○ 1 Oui, indépendamment du COVID-19
  - **Si oui, à quelle fréquence ?**
    - ○ 1 Plusieurs fois par semaine
    - ○ 2 1 fois par semaine
    - ○ 3 1 fois toutes les 2 semaines
    - ○ 4 1 fois par mois
    - ○ 5 Moins d'une fois par mois
- ○ 2 Oui, à cause du COVID-19
  - **Si oui, à quelle fréquence ?**
    - ○ 1 Plusieurs fois par semaine
    - ○ 2 1 fois par semaine
    - ○ 3 1 fois toutes les 2 semaines
    - ○ 4 1 fois par mois
    - ○ 5 Moins d'une fois par mois
- ○ 3 Non

Q24 Au cours des 12 derniers mois, avez-vous aidé une ou des personnes ayant des problèmes de santé, qu'elles vivent ou non avec vous ?
(p.ex. des malades, des personnes handicapées ou des personnes âgées, en les aidant dans leur ménage, en leur apportant à manger ou en effectuant des transports)

- ○ 1 Oui, indépendamment du COVID-19
  - **Si oui, à quelle fréquence ?**
    - ○ 1 Plusieurs fois par semaine
    - ○ 2 1 fois par semaine
    - ○ 3 1 fois toutes les 2 semaines
    - ○ 4 1 fois par mois
    - ○ 5 Moins d'une fois par mois
- ○ 2 Oui, à cause du COVID-19
  - **Si oui, à quelle fréquence ?**
    - ○ 1 Plusieurs fois par semaine
    - ○ 2 1 fois par semaine
    - ○ 3 1 fois toutes les 2 semaines
    - ○ 4 1 fois par mois
    - ○ 5 Moins d'une fois par mois
- ○ 3 Non

Q25 Lorsque vous recevez des informations écrites sur un traitement médical ou sur votre état de santé, avez-vous de la peine à les comprendre ?

- ○ 1 Jamais
- ○ 2 Parfois
- ○ 3 Souvent
- ○ 4 Toujours

Q26 De façon générale, vous diriez que le système de santé en Suisse …

- ○ 1 … ne nécessite aucune réforme
- ○ 2 … nécessite peu de réformes
- ○ 3 … nécessite beaucoup de réformes
- ○ 4 … nécessite une réforme radicale
- ○ 5 Sans avis

Q27 Est-il juste ou injuste que les plus riches puissent s'offrir des soins médicaux de meilleure qualité que les plus pauvres ?

- ○ 1 Tout à fait juste
- ○ 2 Plutôt juste
- ○ 3 Ni juste, ni injuste
- ○ 4 Plutôt injuste
- ○ 5 Tout à fait injuste
- ○ 6 Sans avis

Q28 Dans quelle mesure seriez-vous prêt·e à payer plus d'impôts pour des soins médicaux de meilleure qualité pour toutes et tous en Suisse ?

- ○ 1 Tout à fait prêt·e
- ○ 2 Plutôt prêt·e
- ○ 3 Ni prêt·e, ni pas prêt·e
- ○ 4 Plutôt pas prêt·e
- ○ 5 Pas prêt·e du tout
- ○ 6 Sans avis

Q29 Dans quelle mesure êtes-vous en faveur ou opposé·e à un système d'assurance maladie dans lequel l'assurance de base obligatoire (LAMal) serait publique, c'est-à-dire gérée par la Confédération ou les cantons ?

- ○ 1 Très favorable
- ○ 2 Plutôt favorable
- ○ 3 Ni favorable, ni opposé·e
- ○ 4 Plutôt opposé·e
- ○ 5 Très opposé·e
- ○ 6 Sans avis

Q30 Dans quelle mesure êtes-vous d'accord avec les affirmations suivantes concernant ce questionnaire ?

	Tout à fait d'accord	Plutôt d'accord	Ni d'accord, ni en désaccord	Plutôt en désaccord	Tout à fait en désaccord	Sans avis
Ce questionnaire était trop long	○ 1	○ 2	○ 3	○ 4	○ 5	○ 6
Il était difficile de faire des choix entre les différents scénarios présentés	○ 1	○ 2	○ 3	○ 4	○ 5	○ 6
Les instructions n'étaient pas claires	○ 1	○ 2	○ 3	○ 4	○ 5	○ 6

Q31 Avez-vous d'autres commentaires à faire au sujet du questionnaire ?

✍

Nous vous remercions de nous avoir aidés à mener cette recherche en répondant à notre enquête !

Merci de nous retourner le questionnaire rempli en utilisant l'enveloppe retour préaffranchie jointe à notre courrier.
