# Supplementary material for: Preferences for innovations in healthcare delivery models in the Swiss elderly population: a latent class, choice modelling study
Source: Eur J Public Health. 2024 Jan 18;34(2):260–6. doi: 10.1093/eurpub/ckae004 (PMC10990495; doi:10.1093/eurpub/ckae004)
Supplement: ckae004_Supplementary_Data [file ckae004_supplementary_data.zip › ckae004_Supplementary_Data/ejph-2023-06-om-0339-File004.pdf]

unisanté

Centre universitaire de médecine générale et santé publique · Lausanne

Study on the Swiss healthcare system

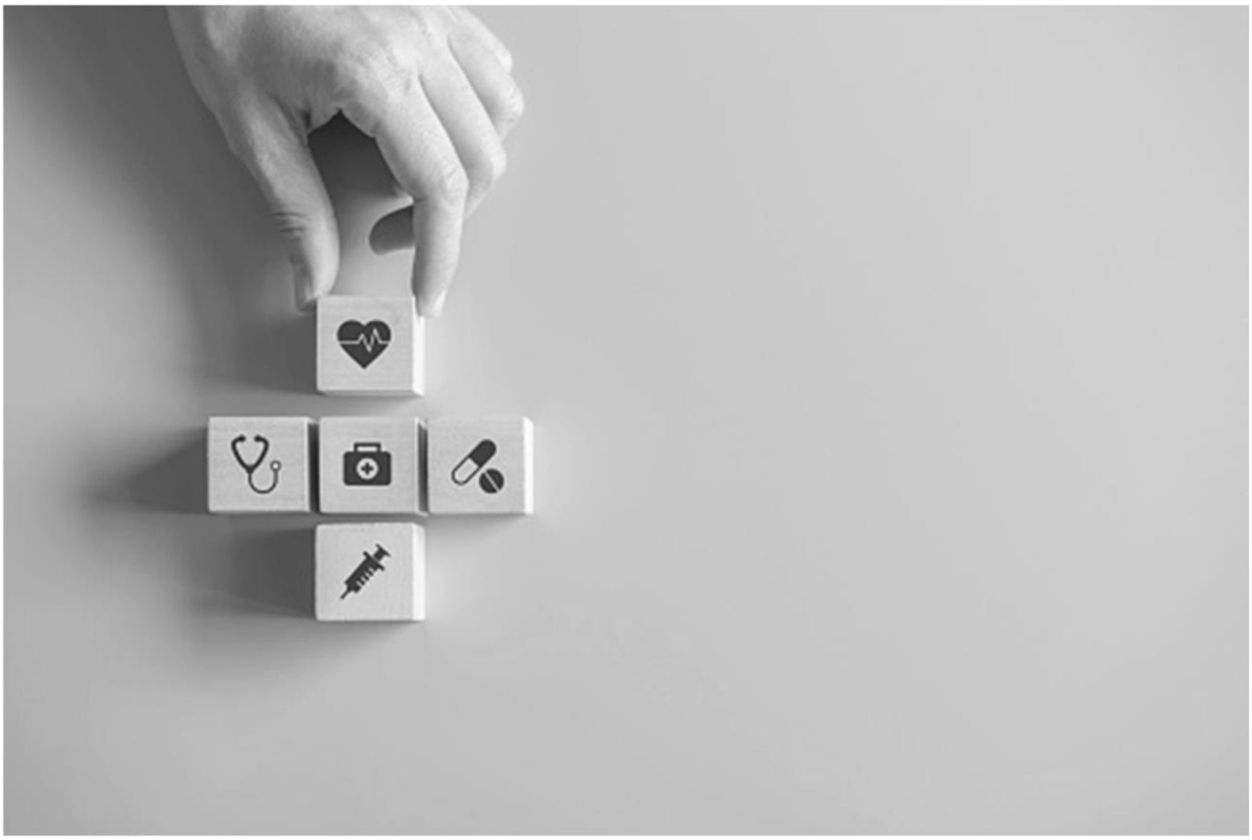

Paper questionnaire
2021

V1XXX

INSTRUCTIONS FOR COMPLETING THE QUESTIONNAIRE

The present survey covers preferences in relation to the healthcare system among the Swiss population aged 50 and over.

The questionnaire takes around 25 minutes to complete. Your data and answers will be treated **confidentially and anonymously**.

Thank you for taking the time to complete our survey.

Here are some brief instructions and details:

- Please use **a dark ballpoint pen** or other clearly legible pen to complete the form.
- Please enter your answer with a cross in the field of your choice: ⊗
- If you have ticked an incorrect box, please cross out the incorrect box, going clearly beyond the edges, and make a new cross in the desired box: ⊗
- Please give **only one answer** per question, except for questions with the remark "Several answers possible". These are identified by square boxes.
- The ☒ symbol indicates a written answer without abbreviations.

If you have any questions, please do not hesitate to contact us:

- Telephone: **021 692 46 72**
- E-mail: **sante2021@fors.unil.ch**

Please return the completed questionnaire using the prepaid envelope enclosed:

FORS - 5005
University of Lausanne
Bâtiment Géopolis
1015 Lausanne

SECTION 1 - GENERAL QUESTIONS

Q1 Are you ?

- ○ 1 A woman
- ○ 2 A man
- ○ 3 Other/non-binary

Q2 What is your year of birth?

✍ | | | | |

Q3 What is your canton of residence in Switzerland?

- ○ 1 Bern (Bernese Jura region)
- ○ 2 Fribourg
- ○ 3 Geneva
- ○ 4 Jura
- ○ 5 Neuchâtel
- ○ 6 Valais (Bas-Valais)
- ○ 7 Vaud
- ○ 8 Other

Q4 What is your marital status?

- ○ 1 Single
- ○ 2 Married / Registered partnership
- ○ 3 Separated / Divorced / Partnership dissolved
- ○ 4 Widow/widower

Q5 Which of the following best describes your current situation?

- ○ 1 Lives alone
- ○ 2 Single-parent family (one parent with child(ren))
- ○ 3 Couple with no children
- ○ 4 Couple with children
- ○ 5 Living in an institution (EMS)
- ○ 6 Other (please specify) ✍

Q6 What is the highest level of training you have completed?

- ○ 1 No completed schooling
- ○ 2 Compulsory school (elementary school, orientation cycle)
- ○ 3 Apprenticeship, vocational training
- ○ 4 Post-compulsory school (e.g. college, business school, school of general education)
- ○ 5 Tertiary education (e.g. university, college)

Q7 Which option best describes your current professional situation?

- ◯ [1] Full-time employee (80-100%)
- ◯ [2] Part-time employee (less than 80%)
- ◯ [3] Self-employed worker
- ◯ [4] Unemployed
- ◯ [5] Retired
- ◯ [6] To disability insurance (AI)
- ◯ [7] At home doing household and family chores
- ◯ [8] Other (please specify) ✍

Q8 Which professional category best corresponds to the activity you are (or were) engaged in?

- ◯ [1] Laborer, worker
- ◯ [2] Skilled worker, foreman
- ◯ [3] Farmer
- ◯ [4] Untrained employee (e.g. office assistant)
- ◯ [5] Qualified employee (e.g. secretary, accountant)
- ◯ [6] Middle management (e.g. technician, teacher)
- ◯ [7] Self-employed small business owner, craftsperson
- ◯ [8] Senior manager (e.g. economist, company lawyer)
- ◯ [9] Liberal profession (e.g. doctor, lawyer)
- ◯ [10] Director, company or public service manager
- ◯ [11] I've never had a job
- ◯ [12] Other (please specify) ✍

Q9 If you add up all your sources of income, what is your total net income per month?

- ◯ [1] Less than CHF 3,000
- ◯ [2] Between CHF 3,000 and CHF 5,000
- ◯ [3] Between CHF 5'001 and CHF 7'000
- ◯ [4] Between CHF 7'001 and CHF 9'000
- ◯ [5] Between CHF 9'001 and CHF 11'000
- ◯ [6] Between CHF 11'001 and CHF 13'000
- ◯ [7] Over CHF 13,000
- ◯ [8] Don't know
- ◯ [9] I do not wish to answer

SECTION 2 - HEALTH SYSTEM PREFERENCES

In this section, we'd like to better understand your preferences regarding the Swiss healthcare system, and identify certain features that are particularly important to you.

To do this, we're going to ask you to make a series of choices for 8 scenarios proposing two options describing access to and use of healthcare in Switzerland.

These 8 scenarios are different, yet similar. The repetition of these scenarios helps us to understand the importance you attach to the different features of the healthcare system.

Each option is described in terms of 6 characteristics, which are explained below.

Please pay particular attention to the following six boxes, as they are extremely important for understanding the rest of the survey.

Please note that some of these characteristics do not reflect Swiss reality, but describe policies that could be implemented in the future.

DESCRIPTION OF THE 6 FEATURES PRESENTED IN THE SCENARIOS: 1/6

Feature 1: Which professionals have access to my electronic patient record?

Explanation: The electronic patient record (EPR) is a secure computerized record that brings together various information concerning your health. Since 2017, a federal law has required hospitals and nursing homes to adopt an EPR; the latter is optional for outpatient care. Access is guaranteed at all times and in all places via a secure Internet connection, and the processing of the information it contains guarantees the confidentiality of your data.

This feature can take the following forms:

A. **My family doctor** only
B. **All the doctors involved** in my care
C. **All healthcare professionals** (doctors and non-physicians) **involved** in my care
D. **All healthcare professionals involved** in my care and **my basic health insurance company**

DESCRIPTION OF THE 6 FEATURES PRESENTED IN THE SCENARIOS: 2/6

Feature 2: What is the variation in my monthly basic health insurance premium?

Explanation: This is the monthly contribution you pay to your basic health insurance fund.

This feature can take the following forms:

A. My monthly premium is reduced by 50 francs (**- 50 CHF**)
B. My monthly premium is reduced by 100 francs (**- 100 CHF**)
C. My monthly premium **remains the same**
D. My monthly premium increases by 50 francs (**+ 50 CHF**)
E. My monthly premium increases by 100 francs (**+ 100 CHF**)

DESCRIPTION OF THE 6 FEATURES PRESENTED IN THE SCENARIOS: 3/6

Feature 3: Which professional(s) is/are clearly designated to coordinate my care?

<u>Explanation</u>: Care coordination is necessary whenever several healthcare professionals are involved in your care. It requires the communication of information between you (the patient) and the professionals involved, with the participation of your family and friends. In addition to communicating information, a professional designated to coordinate your care will have the following tasks: monitor and coordinate your treatment plan, provide information on your state of health, answer your questions, define with you a care or health plan to meet your personal needs, consult and collaborate with other health services, care providers and specialists.

<u>This feature can take the following forms:</u>

A. No clearly designated **healthcare professional**

B. **My family doctor**

C. **A** non-physician **healthcare professional** (e.g. nurse, medical assistant)

D. **A care team** including several healthcare professionals (doctors and non-physicians)

E. A referral (doctor or non-physician) from **my basic health insurance fund**

DESCRIPTION OF THE 6 FEATURES PRESENTED IN THE SCENARIOS: 4/6

Feature 4: How do I access specialists?

<u>Explanation</u>: Today, in the standard basic health insurance model, there are no restrictions on your choice of doctors, and you can go directly to a specialist. With certain alternative insurance models, you must go through your family doctor to be referred to a specialist (with the exception of emergency consultations and consultations with an ophthalmologist or gynecologist).

<u>This feature can take the following forms:</u>

A. **Direct access to** a specialist

B. No possibility of consulting a specialist directly; **my family doctor does** it for me.
who refers me to a specialist

C. I can consult **directly only the specialists** indicated in **a defined list.**
in my basic health insurance contract

DESCRIPTION OF THE 6 FEATURES PRESENTED IN THE SCENARIOS: 5/6

Feature 5: Do chronically ill policyholders pay a deductible and/or co-payment?

<u>Explanation</u>: Currently, as part of their basic health insurance, policyholders must pay a deductible (ranging from CHF 300 to CHF 2,500 for adults) in addition to their monthly premium. Once the deductible has been reached, the insured person pays a co-payment (i.e. a contribution to costs) of 10%, up to a maximum of CHF 700 per year.

<u>This feature can take the following forms:</u>

A. Chronically ill policyholders pay **a deductible and a co-payment.**

B. Chronically ill policyholders pay **only the co-payment**

C. Chronically ill policyholders pay **only the deductible**

D. Chronically ill policyholders pay **neither deductible nor co-payment**

DESCRIPTION OF THE 6 FEATURES PRESENTED IN THE SCENARIOS: 6/6

Characteristic 6: Is care and support for family caregivers compensated?

<u>Explanation</u>: At present, the care and support provided by family caregivers, i.e. people who look after loved ones requiring assistance, whether occasional or permanent, is not formally compensated.

<u>This feature can take the following forms:</u>

A. Care and support are **formally compensated** (e.g. reduced bonus, additional paid leave)

B. Care and support are **formally compensated** and family caregivers have access to **specific services** (e.g. information, training, psychological support)

C. Care and support are **not formally compensated**

For each of the following 8 questions, we ask you to **choose your preferred option** between the two presented.

When you make this choice :

- Carefully consider all six features
- Two out of six features do not differ between options (marked in dark grey).
- Consider that all other health system characteristics (not shown in the table) are the same between the two options.
- Answer all the choices, considering each time that only these two options are available
- There's no right or wrong answer!

On the next page, we show you **an example** of such a scenario.

Which of the following options do you prefer?

	Option 1	Option 2
**Which professionals have access to my electronic patient record?**	My family doctor only	All the healthcare professionals involved and my basic health insurance company
**Who coordinates my care?**	A care team	No healthcare professionals
**Access to specialists**	**Direct consultation available (free choice)**	**Direct consultation available (free choice)**
**What do chronically ill policyholders pay?**	No deductible or co-payment	Only co-payment
**Formal compensation for care and support of family caregivers**	Yes	No
**Change in my monthly basic health insurance premium**	**+ CHF 50**	**+ CHF 50**

	Option 1	Option 2
Your choice :	○ 1	⊗ 2

	Your current situation	The option selected above
If you could now keep your current situation : what would you choose?	○ 1	⊗ 2

In this example, the person prefers option 2 to option 1 and to the current situation.

From now on, we'll ask you to make a series of choices similar to the previous example.

If you need to, you can reread the feature definitions (presented on pages 5 to 7) when responding to the various scenarios.

Scenario 1: Which of the following options do you prefer?

	Option 1	Option 2
**Which professionals have access to my electronic patient record?**	My family doctor only	All the healthcare professionals involved and my basic health insurance company
**Who coordinates my care?**	A care team	No healthcare professional
**Access to specialists**	**Direct consultation available (free choice)**	**Direct consultation available (free choice)**
**What do chronically ill policyholders pay?**	No deductible or co-payment	Only co-payment
**Formal compensation for care and support for family caregivers**	Yes	No
**Change in my monthly basic health insurance premium**	**+50 CHF**	**+50 CHF**

	**Option 1**	**Option 2**
Your choice :	○ 1	○ 2

	**Your current situation**	**The option selected above**
If you could now keep your current situation : what would you choose?	○ 1	○ 2

Scenario 2: Which of the following options do you prefer?

	Option 1	Option 2
**Which professionals have access to my electronic patient record?**	**My family doctor only**	**My family doctor only**
**Who coordinates my care?**	My family doctor	A referral from my basic health insurance fund
**Access to specialists**	Direct consultation possible if doctor is on a list (restricted choice)	Need to be referred by the family doctor (gatekeeping)
**What do chronically ill policyholders pay?**	Only co-payment	No deductible or co-payment
**Formal compensation for care and support of family caregivers**	No	Yes, and access to specific support services
**Change in my monthly basic health insurance premium**	**-50 CHF**	**-50 CHF**

	**Option 1**	**Option 2**
Your choice :	○ 1	○ 2

	**Your current situation**	**The option selected above**
If you could now keep your current situation : what would you choose?	○ 1	○ 2

Scenario 3: Which of the following options do you prefer?

	Option 1	Option 2
**Which professionals have access to my electronic patient record?**	**All the healthcare professionals involved and my basic health insurance company**	**All the healthcare professionals involved and my basic health insurance company**
**Who coordinates my care?**	My family doctor	A non-physician healthcare professional
**Access to specialists**	Direct consultation possible if the doctor is on a list (restricted choice)	Direct consultation available (free choice)
**What do chronically ill policyholders pay?**	Only co-payment	A deductible and a co-payment
**Formal compensation for care and support of family caregivers**	Yes, and access to specific support services	No
**Change in my monthly basic health insurance premium**	**-100 CHF**	**-100 CHF**

	**Option 1**	**Option 2**
Your choice :	○ 1	○ 2

	**Your current situation**	**The option selected above**
If you could now keep your current situation : what would you choose?	○ 1	○ 2

Scenario 4: Which of the following options do you prefer?

	Option 1	Option 2
**Which professionals have access to my electronic patient record?**	My family doctor only	All the healthcare professionals involved and my basic health insurance company
**Who coordinates my care?**	No healthcare professional	A non-physician healthcare professional
**Access to specialists**	Direct consultation available (free choice)	Need to be referred by the family doctor (gatekeeping)
**What do chronically ill policyholders pay?**	**Only co-payment**	**Only co-payment**
**Formal compensation for care and support for family caregivers**	No	Yes, and access to specific support services
**Change in my monthly basic health insurance premium**	**+100 CHF**	**+100 CHF**

	**Option 1**	**Option 2**
Your choice :	○ 1	○ 2

	**Your current situation**	**The option selected above**
If you could now keep your current situation : what would you choose?	○ 1	○ 2

Scenario 5: Which of the following options do you prefer?

	Option 1	Option 2
**Which professionals have access to my electronic patient record?**	My family doctor only	All the doctors involved in my care
**Who coordinates my care?**	A care team	A non-physician healthcare professional
**Access to specialists**	Direct consultation available (free choice)	Direct consultation possible if the doctor is on a list (restricted choice)
**What do chronically ill policyholders pay?**	**A deductible and a co-payment**	**A deductible and a co-payment**
**Formal compensation for care and support for family caregivers**	**No**	**No**
**Change in my monthly basic health insurance premium**	-50 CHF	-100 CHF

	**Option 1**	**Option 2**
Your choice :	○ 1	○ 2

	**Your current situation**	**The option selected above**
If you could now keep your current situation : what would you choose?	○ 1	○ 2

Scenario 6: Which of the following options do you prefer?

	Option 1	Option 2
**Which professionals have access to my electronic patient record?**	All the doctors involved in my care	All the healthcare professionals involved and my basic health insurance company
**Who coordinates my care?**	My family doctor	A care team
**Access to specialists**	**Need to be referred by the family doctor (gatekeeping)**	**Need to be referred by the family doctor (gatekeeping)**
**What do chronically ill policyholders pay?**	Franchise only	Only co-payment
**Formal compensation for care and support for family caregivers**	**No**	**No**
**Change in my monthly basic health insurance premium**	+50 CHF	+100 CHF

	**Option 1**	**Option 2**
Your choice :	○ 1	○ 2

	**Your current situation**	**The option selected above**
If you could now keep your current situation : what would you choose?	○ 1	○ 2

Scenario 7: Which of the following options do you prefer?

	Option 1	Option 2
**Which professionals have access to my electronic patient record?**	All the doctors involved in my care	All the healthcare professionals involved in my care
**Who coordinates my care?**	A care team	My family doctor
**Access to specialists**	**Need to be referred by the family doctor (gatekeeping)**	**Need to be referred by the family doctor (gatekeeping)**
**What do chronically ill policyholders pay?**	Franchise only	A deductible and a co-payment
**Formal compensation for care and support of family caregivers**	**Yes, and access to specific support services**	**Yes, and access to specific support services**
**Change in my monthly basic health insurance premium**	-100 CHF	+50 CHF

	**Option 1**	**Option 2**
Your choice :	○ 1	○ 2

	**Your current situation**	**The option selected above**
If you could now keep your current situation : what would you choose?	○ 1	○ 2

Scenario 8: Which of the following options do you prefer?

	Option 1	Option 2
**Which professionals have access to my electronic patient record?**	My family doctor only	All the doctors involved in my care
**Who coordinates my care?**	A care team	A non-physician healthcare professional
**Access to specialists**	Direct consultation available (free choice)	Direct consultation possible if doctor is on a list (restricted choice)
**What do chronically ill policyholders pay?**	**A deductible and a co-payment**	**A deductible and a co-payment**
**Formal compensation for care and support for family caregivers**	**No**	**No**
**Change in my monthly basic health insurance premium**	-50 CHF	-100 CHF

	**Option 1**	**Option 2**
Your choice :	○ 1	○ 2

	**Your current situation**	**The option selected above**
If you could now keep your current situation : what would you choose?	○ 1	○ 2

SECTION 3 - MEDICAL SITUATION AND USE OF THE HEALTHCARE SYSTEM

We'd now like to ask you a few questions about your medical situation and your use of the healthcare system.

Q10 How would you describe your general state of health?

1 Very good

2 Good

3 Neither good nor bad

4 Bad

5 Very bad

Q11 Over the past 12 months, have you been treated for, or suffered from, any of the following health problems diagnosed by a doctor?

Check all that apply to you, multiple answers possible

- ☐ 1 High blood pressure (hypertension)
- ☐ 2 High blood cholesterol (fats)
- ☐ 3 Angina pectoris or angina, myocardial infarction, heart attack (coronary problem)
- ☐ 4 Heart failure, valve disease or heart muscle disease
- ☐ 5 Stroke (ictus, stroke)
- ☐ 6 Diabetes (type I or type II)
- ☐ 7 Chronic lung disease (asthma, chronic bronchitis, COPD) chronic lung disease), emphysema)
- ☐ 8 Osteoporosis
- ☐ 9 Osteoarthritis or arthritis
- ☐ 10 Cancer, malignant tumor, lymphoma
- ☐ 11 Stomach and duodenal ulcers, peptic ulcers
- ☐ 12 Inflammatory bowel disease (Crohn's disease, ulcerative colitis)
- ☐ 13 Depression
- ☐ 14 Parkinson's disease, Alzheimer's disease
- ☐ 15 HIV infection
- ☐ 16 Covid-19
- ☐ 17 None of these health problems

Q12 Which health insurance model do you personally use for compulsory basic insurance?

- ◯ 1 Ordinary insurance model
- ◯ 2 Health insurance network model (HMO)
- ◯ 3 Family doctor model
- ◯ 4 Model providing for a medical consultation by telephone before/before each medical check-up
- ◯ 5 Other (please specify) ✍
- ◯ 6 I don't know

Q13 What is your personal annual deductible?
The deductible is the portion of your medical expenses that you must pay (not covered by insurance).

- ◯ 1, CHF 300
- ◯ 2 CHF 500
- ◯ 3 CHF 1,000
- ◯ 4 CHF 1,500
- ◯ 5 CHF 2,000
- ◯ 6 CHF 2,500
- ◯ 7 I don't know

Q14 What is your monthly premium for basic insurance?
(please do not take into account state aid/subsidies if you receive them)

- ◯ 1 Less than CHF 200
- ◯ 2,200 - 250 CHF
- ◯ 3 251 - 300 CHF
- ◯ 4 301 - 350 CHF
- ◯ 5, 351 - 400 CHF
- ◯ 6 401 - 450 CHF
- ◯ 7, 451 - 500 CHF
- ◯ 8 501 - 550 CHF
- ◯ 9,551 - 600 CHF
- ◯ 10 Over CHF 600
- ◯ 11 I don't know

Q15 Do you currently receive subsidies from the canton for your health insurance premiums?

- ○1 Yes
- ○2 No
- ○3 I don't know

Q16 Do you have at least one supplementary insurance policy (e.g. complementary medicine, dental care, hospitalization, glasses, coverage abroad)?

- ○1 Yes
- ○2 No
- ○3 I don't know

Q17 When it comes to choosing a basic health insurance contract, are you confident in your ability to select the one that suits you best?

- ○1 Very confident
- ○2 Somewhat confident
- ○3 Neither confident nor not confident
- ○4 Somewhat not confident
- ○5 Not at all confident

Q18 In the past 12 months, have you ever had to forego certain health care services for financial reasons?

- ○1 Yes
- ○2 No

Q19 Do you have a family doctor or personal GP?
The family doctor is the doctor you can go to for most of your health problems.

- ○1 Yes

  ↳ **In the last 12 months, how many times in total have you visited your family doctor, or another general practitioner?**

  Number of times (in figures) ✍ ____

- ○2 No
- ○3 I don't know

Q19 In the last 12 months, have you consulted a specialist *(including gynecologist and ophthalmologist, but not including dentists)*?

○ 1 Yes

↳ **In the last 12 months, how many times in total have you visited a specialist *(including gynecologists and ophthalmologists, but excluding dentists)*?**

Number of times (in figures) ✍

○ 2 No

Q20 In the last 12 months, have you been hospitalized?
Take into account stays in medical, surgical, psychiatric or other specialized departments.

○ 1 Yes

↳ **In total, how many nights have you spent in hospital in the last 12 months?**

Number of nights (in figures) ✍

○ 2 No

Q21 In the last 12 months, have you consulted a hospital emergency department directly, without being referred by a doctor or ambulance?

○ 1 Yes

↳ **How many times have you consulted a hospital emergency department directly?**

Number of times (in figures) ✍

○ 2 No

Q22 In the last 12 months, have you spent one or more nights in a nursing home?

○ 1 Yes

○ 2 No

Q23 In the last 12 months, have you received help with your shopping, meals, care or administrative tasks for health reasons?

○ 1 Yes, independent of COVID-19

↳ **If so, how often?**

○ 1 Several times a week
○ 2 1 time per week
○ 3 1 time every 2 weeks
○ 4 1 time per month
○ 5 Less than once a month

○ 2 Yes, because of COVID-19

↳ **If so, how often?**

○ 1 Several times a week
○ 2 1 time per week
○ 3 1 time every 2 weeks
○ 4 1 time per month
○ 5 Less than once a month

○ 3 No

Q24 In the last 12 months, have you helped anyone with health problems, whether or not they live with you?
(e.g. the sick, disabled or elderly, by helping them with housework, bringing them food or providing transport)

○ 1 Yes, independent of COVID-19

↳ **If so, how often?**

○ 1 Several times a week
○ 2 1 time per week
○ 3 1 time every 2 weeks
○ 4 1 time per month
○ 5 Less than once a month

○ 2 Yes, because of COVID-19

↳ **If so, how often?**

○ 1 Several times a week
○ 2 1 time per week
○ 3 1 time every 2 weeks
○ 4 1 time per month
○ 5 Less than once a month

○ 3 No

Q25 When you receive written information about a medical treatment or your state of health, do you find it difficult to understand?

- ○ 1 Never
- ○ 2 Sometimes
- ○ 3 Often
- ○ 4 Always

Q26 In general, would you say that the Swiss health care system …

- ○ 1 ... requires no reform
- ○ 2 ... requires few reforms
- ○ 3 ... requires many reforms
- ○ 4 ... requires radical reform
- ○ 5 No opinion

Q27 Is it fair or unfair that the richest can afford better medical care than the poorest?

- ○ 1 Quite right
- ○ 2 Somewhat right
- ○ 3 Neither fair nor unfair
- ○ 4 Somewhat unfair
- ○ 5 Quite unfair
- ○ 6 No opinion

Q28 To what extent would you be prepared to pay higher taxes for better medical care for everyone in Switzerland?

- ○ 1 Completely ready
- ○ 2 Somewhat ready
- ○ 3 Neither ready nor not ready
- ○ 4 Somewhat not ready
- ○ 5 Not ready at all
- ○ 6 No opinion

Q29 To what extent do you support or oppose a health insurance system in which compulsory basic insurance (KVG/LAMal) would be public, i.e. managed by the Confederation or the cantons?

- ○1 Very much support
- ○2 Rather support
- ○3 Neither support nor oppose
- ○4 Rather oppose
- ○5 Very much oppose
- ○6 No opinion

Q30 To what extent do you agree with the following statements regarding this survey?

	Very much agree	Rather agree	Neither agree nor disagree	Rather disagree	Very much disagree	No opinion
This survey is too long	○1	○2	○	○4	○	○
It was difficult to make a choice between the presented scenarios	○1	○2	○	○4	○5	○
The instructions are unclear	○1	○2	○3	○4	○5	○6

Q31 Do you have any other comments about the questionnaire?

✍
